# Formulation of Sodium Valproate Nanospanlastics as a Promising Approach for Drug Repurposing in the Treatment of Androgenic Alopecia

**DOI:** 10.3390/pharmaceutics12090866

**Published:** 2020-09-11

**Authors:** Farid. A. Badria, Hassan A. Fayed, Amira K. Ibraheem, Ahmed F. State, Eman A. Mazyed

**Affiliations:** 1 Department of Pharmacognosy, Faculty of Pharmacy, Mansoura University, Mansoura 33516, Egypt; 2 Department of Dermatology, Faculty of Medicine, Mansoura University, Mansoura 33516, Egypt; fayedha2004@mans.edu.eg (H.A.F.); ahmedstate@mans.edu.eg (A.F.S.); 3 Mansoura Dermatology and Leprosy Hospital, Mansoura 35511, Egypt; kotbamira11@gmail.com; 4 Department of Pharmaceutical Technology, Faculty of Pharmacy, Kafrelsheikh University, Kafrelsheikh 33516, Egypt; eman_mazyad@pharm.kfs.edu.eg

**Keywords:** sodium valproate, nanospanlastics, edge activators, topical delivery, androgenic alopecia

## Abstract

Sodium valproate (SV) is an antiepileptic drug that is widely used in the treatment of different seizure disorders. The topical SV has a hair regenerative potential through activating the Wnt/β-catenin pathway and anagen phase induction. The aim of the current investigation was to fabricate nanospanlastics of SV for improving its dermal delivery by providing prolonged drug effect and increasing its permeability for treatment of androgenic alopecia (AGA). SV-loaded nanospanlastics were formulated according to 2^3^ factorial design by ethanol injection method using a non-ionic surfactant (Span 60) and edge activators (EAs), such as Tween 80 and Cremophor RH 40, to explore the influence of different independent variables on entrapment efficiency (EE%) and percentage drug released after 12 h (Q_12h_) in order to choose the optimized formula using Design-Expert software. The optimized formula (F8) appeared as spherical deformable vesicles with EE% of 90.32 ± 2.18% and Q_12h_ of 90.27 ± 1.98%. F8 exhibited significant improvement of ex vivo permeation than free SV. The clinical study exhibited no comparable difference between F8 and marketed minoxidil lotion. However, F8 demonstrates less adverse effects than minoxidil lotion. Nanospanlastics could be a safe and effective method for improving the topical delivery of SV in the management of AGA.

## 1. Introduction

Androgenic alopecia (AGA) is a progressive hair loss condition that may be attributed to androgen-mediated thinning of scalp hair by conversion of susceptible terminal hair to vellus hair [1]. It is also known as patterned hair loss that affects both males (male patterned hair loss) and females (female patterned hair loss) [2]. There is a strong correlation between family history and AGA in relation to its severity and time of onset. The pathophysiology of AGA involves shortening of the hair cycle, increasing the telogen shedding and elongation of the catagen phase duration [3].

To date, many studies have been performed using different therapies, such as minoxidil and finasteride. However, these therapies have a number of side effects that have a great impact on patient compliance and their adherence to therapy. Minoxidil is an antihypertensive agent that is used for the treatment of AGA by prolongation of the anagen phase as well as increasing the vasculature of hair follicles. Unfortunately, minoxidil has a number of unwanted effects, such as facial hypertrichosis, scalp irritation, scaling, and redness [4,5]. Concerning finasteride, its oral administration decreases dihydrotestosterone serum concentrations and increase the number of terminal hairs in the anagen phase by preventing the miniaturization of the hair follicles. However, it causes unwanted effects including erectile dysfunction, ejaculation disorder and decreased libido [6].

Sodium valproate (SV), chemically designated as 2-propyl pentanoate, is the sodium salt of valproic acid (VA). SV is a histone deacetylase inhibitor that is widely used in the treatment of epilepsy, migraine, bipolar disorders, and different seizure disorders [7]. The common side effects of oral valproate administration involve weight gain, tremor, liver dysfunction, gastrointestinal disturbances, thrombocytopenia, metabolic acidosis, and hair loss. The progressive increase in the dose is considered to be a critical factor in counteracting SV-induced hair loss [8]. In contrast to the oral administration of SV that causes hair loss, topical delivery of valproate showed effective hair regeneration [9]. The hair regenerative effect of SV is due to anagen phase induction and activation of the Wnt/β-catenin pathway that is important for the differentiation of stem cells into follicular keratinocytes and the development of hair follicles [10]. Hence, SV could be repurposed for treatment of AGA.

A limited number of studies investigate the efficacy of topical VA and SV for treating AGA. Jo et al. [11] performed a comparative clinical study for male patients with moderate AGA using phototrichogram analysis. The results showed that topical SV could effectively increase the total hair count. Choi et al. [12] explored that VA has a potential effect on the hair cycle and could be used to treat AGA. Lee et al. [10] reported that the topical application of VA stimulated hair regrowth in mice and activated alkaline phosphatase activity in human dermal papilla cells by activating the Wnt/β-catenin pathway. These studies verify the potential of VA and SV to be used for hair growth. However, these studies do not discuss how to improve the dermal delivery and permeability of these drugs.

For dermal drug delivery, the major challenge is the barrier behavior of the outer layer of skin (stratum corneum (SC)) that limits drug permeation and hinders attaining reasonable therapeutic concentrations. A large number of techniques have been performed to overcome the low permeability of SC in order to increase drug concentrations in the hair follicle [13]. Nano-vesicular systems like liposomes and niosomes are considered to be effective matrices for bioencapsulation of many drugs. In recent years, nanospanlastics received great attention as a potential drug delivery system that has higher elasticity and permeability through different skin layers than conventional vesicles. Nanospanlastics are elastic surfactant-based vesicles that consist mainly of non-ionic surfactants and edge activators (EAs) [14]. The deformability of nanospanlastic vesicles are mainly due to the incorporation of EAs. They could fluidize the vesicular bilayer by decreasing its interfacial tension and thus improve drug permeation across the biological membranes [15]. Compared to phospholipid nanovesicles, spanlastics offer higher physical and chemical stability. In addition, these deformable nanovesicles are more advantageous than niosomes because they provide a greater ability not only to overcome the SC barrier but also to penetrate efficiently through different skin layers by squeezing through different pores and channels that are smaller than their size. Therefore, spanlastics have been reported to enhance the topical delivery of many drugs and increase the residence time of drugs within different skin layers [14,16]. The objective of this study was to prepare topical nanospanlastics of SV for enhancing its dermal delivery and permeability, thus increasing its therapeutic efficacy in the treatment of AGA and improving patient compliance.

## 2. Materials and Methods

### 2.1. Materials

SV was kindly provided by Western pharmaceutical company (Cairo, Egypt). Sorbitan monostearate (Span 60) was obtained from PureLab, USA. Polyoxyethylene (20) sorbitan monooleate (Tween80) and PEG-40 hydrogenated castor oil (Cremophor RH 40) were purchased from Sigma Chemical Co. (St. Louis, MO, USA). Monobasic potassium phosphate and dibasic potassium phosphate were purchased from Alpha Chemica (Mumbai, India). Absolute ethanol was obtained from El-Nasr Pharmaceutical Chemical Company (Cairo, Egypt). Spectra/Pore^®^ dialysis membrane (12,000–14,000 Mwt cut-off) was purchased from Spectrum Laboratories Inc. (Irving, TX, USA). Acetonitrile (HPLC grade) and phosphoric acid (HPLC grade) were purchased from Sigma-Aldrich Chemical Co. (St. Louis, MO, USA). All other chemicals were of analytical grade and used as received. Marketed 5% minoxidil lotion (Hair Plus Back^®^) was purchased from Minapharm Pharmaceuticals (Cairo, Egypt).

### 2.2. Methods

#### 2.2.1. Preparation of SV-Loaded Spanlastic Nanovesicles (SNVs)

A Preliminary screening test was conducted in order to choose the most suitable levels of different variables for the preparation of SV-loaded SNVs. A total of 27 SV-loaded SNVs were fabricated using three types of EAs (Tween 80, Cremphor RH40, and Brij 35) at different Span 60 to EA ratios (50:50, 60:40, and 80:20) at three different rotation speeds (500, 1000, and 1500) (Supplementary Data, Table S1). Different SV-loaded SNVs were assessed for their EE%. The most suitable two levels were selected for the fabrication of SV-loaded SNVs using Design-Expert^®^ software according to 2^3^ factorial design. All excipients used for preparing SNVs are generally recommended as safe (GRAS) [17] and FDA approved excipients [18]. Span 60 was selected as the membrane-forming lipid due to the high encapsulation efficiency and high stability of Span 60-based vesicles that may be attributable to its lipophilicity and its saturated long (C16) alkyl chain. In addition, it has a high transition temperature (Tc~50 °C) leading to a less leaky vesicular membrane [19]. A number of non-ionic SAAs were used as EAs. Non-ionic surfactants have superior advantages over other types of surfactants with respect to safety, compatibility, and stability [20]. EAs could increase the elasticity of the nanospanlastic vesicles forming systems able to squeeze themselves through different pores of the skin [21].

According to the preliminary screening studies, the most suitable levels of different variables were chosen for the fabrication of SV-loaded SNVs. Eight formulations were prepared according to 2^3^ factorial design using Design-Expert^®^ software, Version 7.0.0 (Stat-Ease, Inc., Minneapolis, MN, USA) in order to explore the influence of different independent variables on the characteristics of spanlastic vesicles [14]. In this model, three different factors were evaluated each at two levels. The independent variables were the ratio of Span 60 to EA (X1), the type of EA (X2) and the rotation speed (X3), whereas %EE (Y1) and % SV released after 12 h (Q_12h_) (Y2) were chosen as the dependent variables (Table 1). The statistical analysis of the results was conducted using the analysis of variance (ANOVA) to determine whether the independent variable has a significant effect on different responses or not. Moreover, the fitness of the present model to the experimental results was explored by estimating determination (R^2^), predicted R^2^ and adjusted R^2^ [22].

The composition of different SV-loaded SNVs is displayed in Table 1. Eight SV-loaded nanospanlastics were fabricated according to the ethanol injection method. The ethanol injection method was adopted for the fabrication of SV-loaded nanospanlastics. It is a simple and reproducible method that could develop small SNVs by injecting the ethanolic solution of the non-ionic surfactant into an aqueous solution of EA [23]. Accurate weights of SV and Span 60 were dissolved into absolute ethanol (2 mL) and injected dropwise into a preheated aqueous solution (70 °C) of the selected EA under magnetic stirring (Jenway 1000, Jenway, UK) till the formation of a milky dispersion of nanospanlastic vesicles. The final volume of the nanospanlastic dispersion was 10 mL. The dispersion was stirred for another 1 h to ensure complete removal of any remaining alcohol. The formed suspension was then exposed to water-bath ultrasonication (Elmasonic E 30 H, Elma, Singen, Germany) for 3 min to obtain an appropriate vesicle size. The formulations were then kept at 4 °C overnight for the maturation of the formed nanospanlastic vesicles to be used for further studies [21].

#### 2.2.2. In Vitro Characterization of SV-Loaded SNVs

##### Determination of Entrapment Efficiency of SV-Loaded SNVs

The entrapment efficiency (EE%) of SV-loaded nanospanlastics was estimated by the indirect method using ultracentrifugation. SV-loaded nanospanlastic dispersion (1mL) was centrifuged using a cooling centrifuge (Hermle Labortechnik GmbH, Wehingen, Germany) at 4 °C for 1 h at 15,000 rpm [16]. The concentration of free SV, in the supernatant, was measured by HPLC at 220 nm. The encapsulation efficiency of SV-loaded SNVs was calculated as follows:

(1) $$\mathrm{EE}\left(\%\right)=\frac{\left(\mathrm{Total}\;\mathrm{amount}\;\mathrm{of}\;\mathrm{SV}-\mathrm{Amount}\;\mathrm{of}\;\mathrm{free}\;\mathrm{SV}\right)\times 100}{\mathrm{Total}\;\mathrm{amount}\;\mathrm{of}\;\mathrm{SV}}$$

##### In Vitro Release Study of SV-Loaded SNVs

The in vitro release of SV-loaded SNVs was compared with the aqueous dispersion of SV to investigate the effect of encapsulation of SV into nanospanlastic vesicles on its in vitro release profile. Prior to performing the test, the solubility of SV was determined in phosphate buffer (pH = 7.4), using the shake-flask method [24] to ensure retaining sink conditions of the selected dissolution medium.

The in vitro release of SV-loaded nanospanlastics was implemented using the membrane diffusion technique using a glass cylinder which is attached to the USP dissolution apparatus shaft (USP apparatus II, Erweka DT-720, Germany) [20,25]. Firstly, the semi-permeable cellulose membrane was hydrated using phosphate buffer (pH = 7.4) for 24 h at room temperature. The presoaked semi-permeable membrane was carefully fixed to the cylinder using a rubber band. An accurate volume (1 mL) of SV-loaded nanospanlastic dispersions containing entrapped SV or the equivalent concentration of SV aqueous dispersion were placed in the donor partition over the semi-permeable membrane. The receptor partition contained 100 mL of phosphate buffer solution (pH = 7.4) as a receptor medium [26,27] which rotated at 100 rpm at 32 ± 0.5 °C during the study to mimic the temperature of the scalp skin [28,29]. A total of 1 mL of aliquot was withdrawn at different time points and replaced by the same volume of phosphate buffer to maintain sink conditions and constant volume of the receptor medium. The results were expressed as mean values ± SD (*n* = 3 per formula). Samples were analyzed using HPLC at 220 nm.

##### Statistical Optimization of SV-Loaded SNVs

Design Expert^®^ software was used to choose the optimal SV-loaded SNVs from the eight nano-spanlastic formulations by the determination of the desirability values. These values refer to the closeness of different responses to their optimal values [30]. The formula that has the highest desirability value is chosen as the optimized formula. The optimized SV-loaded nanospanlastic formula was selected on the basis of maximum EE% and maximum Q_12h_. In addition, the actual values of both EE% and Q_12h_ were compared with their predicted values to validate the optimized formula by calculating the % relative error as follows [31]:

(2) $$\%\;\mathrm{Relative}\;\mathrm{error}=\;\frac{\left(\mathrm{predicted}\;\mathrm{value}-\mathrm{observed}\;\mathrm{value}\right)\times 100\;}{\mathrm{predicted}\;\mathrm{value}}$$

Further studies were performed for the characterization of the optimized nanospanlastic formula.

#### 2.2.3. Characterization of the Optimized SV-Loaded SNVs

##### Morphological Characterization by Scanning Electron Microscopy (SEM)

The morphological features of the optimized SV-loaded nanospanlastic formula were described by scanning electron microscope (JEOL, JSM-6360, Tokyo, Japan). The optimized nanospanlastic dispersion was suitably diluted with deionized water and mounted onto the SEM sample aluminum stub using double-sided sticking carbon tape. The sample was subsequently dried under vacuum and coated with gold film. After coating, the SV-loaded SNVs were examined by SEM [32].

##### Fourier Transform Infrared Spectroscopy (FTIR)

The FTIR spectra were recorded using the FTIR spectrometer (FT-IR Shimadzu 8300 Japan) using potassium bromide (KBr) pellet method. Different samples (3 mg) of SV, Span 60, the chosen EA, the optimized nanospanlastic formula, and the corresponding plain (drug-free) spanlastic formula were mixed with KBr in a hydraulic press (Kimaya Engineers, Thane, Maharastra, India) forming KBr pellets. The scanning range was 4000–400 cm^−1^ [33].

##### Differential Scanning Calorimetry (DSC) Study

Different samples (3 mg) of SV, Span 60, the chosen EA, the optimized nanospanlastic formula, and the corresponding plain (drug-free) spanlastic formula were heated into sealed aluminum pans from 0 to 260 °C with a scanning rate of 10 °C/min [33]. The DSC thermograms were determined using a Shimadzu DSC 60 (Kyoto, Japan).

##### Determination of Particle Size and Zeta Potential of SV-Loaded SNVs

The optimized SV-loaded nanospanlastic formula was suitably diluted by deionized water in order to attain a reasonable scattering intensity. The particle size and zeta potential were determined by a light scattering technique using a NICOMP 380 ZLS Zeta Potential/Particle Sizer (PSS Nicomp, Santa Barbara, CA, USA) by detecting the electrophoretic mobility of the spanlastic vesicles in the electric field. Zeta potential is an important indicator of the stability of the nanospanlastic dispersion by describing the degree of repulsion between particles. Different measurements of both the particle size and zeta potential were done in triplicate and the average of three runs was calculated [21,34].

##### Measurement of Vesicle Elasticity

The elasticity of the optimized SV-loaded SNVs was demonstrated in terms of deformability index (DI) by extrusion of the spanlastic formula through a 100 nm nylon membrane filter for 5 min at 2.5 bar [35]. DI of the optimized SNVs was calculated using the following equation:

(3) $$DI=J\;{\left(r_{v}/r_{p}\right)}^{2}$$

where *J* is the amount of the extruded formulation, *r_v_*: the vesicle size of the optimized SV-loaded SNVs (after extrusion) and *r_p_*: the pore size of the membrane filter.

The deformability of the optimized SV-loaded nanospanlastic dispersion (F8) was compared with the corresponding niosomal dispersion in order to explore the influence of adding EAs on the elasticity of SNVs. The niosomal dispersion was formulated using the ethanol injection method, with the same technique described for preparing spanlastics, using Span 60 and cholesterol at 80:20 ratio and without the addition of EAs [36].

##### Comparative Ex Vivo Permeation between the Optimized SV-Loaded SNVs and Free Drug

The protocol of studying the comparative ex vivo permeation study, of the optimized SV-loaded SNVs, was approved by the ethical committee of the Faculty of Pharmacy, Kafrelsheikh University, Egypt (Approval number KFS-2019/01). Male healthy Wistar rats (200 g) were obtained from the Experimental Animal Center (The National Research Center, Dokki, Giza, Egypt). The rats were allocated in sawdust bed cages at 25 ± 2 °C, relative humidity of 50%, and under 12-h of light/dark cycle. Animal rooms were kept under pathogen-free conditions. Food and water were provided ad libitum. Prior to the experiments, the rats were acclimatized for two weeks in the animal house under standard conditions [33]. The experimental technique was conducted according to the guidelines of the U.K. Animals (Scientific Procedures) Act, 1986 (ASPA) [37], European Union Directive as 2010/63/EU [38] and the ARRIVE guidelines [39].

The ex vivo permeation of the optimized SV-loaded SNVs through rat skin was compared with the aqueous dispersion of SV. The abdominal skin of Wistar albino rats was excised and shaved carefully using a razor to avoid any skin abrasion. The skin integrity was confirmed by inspection under an optical microscope (Coslabs micro, India). The subcutaneous tissue was detached and the dermis side was wiped with isopropyl alcohol to eliminate any adhering fats. The dehaired rat skin was then washed with distilled water and soaked for 2 h in phosphate buffer (pH = 7.4) before performing the experiment.

The comparative ex vivo permeation study of the optimized SV-loaded SNVs was done using the membrane diffusion technique previously described except for the use of the pre-soaked rat skin instead of the dialysis membrane [20,25]. The pre-soaked rat skin was mounted carefully at the cylinder base with the SC side facing the donor chamber. The receiving medium was 100 mL phosphate buffer (pH 7.4) [26,27] that agitated at 100 rpm and maintained at 32 ± 0.5 °C [40]. Afterward, an exact volume (1 mL) of the tested formulation, either the optimized nanospanlastic dispersion or SV dispersion, was loaded in the donor chamber. A total of 1 mL of aliquot was withdrawn from the receiver partition at predetermined time points and replaced with the same volume of phosphate buffer solution [41]. The comparative ex vivo permeation test was performed in triplicate and the results were calculated as mean ± SD. Samples were analyzed using HPLC at 220 nm to determine the amount of SV permeated through rat skin.

The steady-state flux of SV (Jss) was determined from the slope of the linear portion of the plot of cumulative SV permeated through rat skin versus time in steady-state conditions. Permeability coefficient (Kp) was also determined by dividing Jss on the initial concentration of SV. The enhancement ratio (ER) was calculated by dividing Jss of the optimized nanospanlastic formula on Jss of SV aqueous dispersion [14].

#### 2.2.4. Comparative Study between the Optimized SV-Loaded SVs and Marketed Minoxidil Lotion by Dermoscopic Evaluation for the Treatment of Androgenic Alopecia

An intervention randomized comparative study was performed between the optimized SV-loaded spanlastics and marketed 5% minoxidil lotion (Hair Plus Back^®^) at the outpatient clinic of the Department of Dermatology, STDs Department, Mansoura University Hospitals between August 2019 and February 2020.

##### Ethical Consideration

The whole study design was approved by the institutional review board (IRB), Faculty of Medicine, Mansoura University (code number–MS.19.03.544; dated 24 March 2019). The study was conducted in accordance with the ethical guidelines for medical researches involving humans such as the European Directive for clinical research [42] and the Code of Ethics of the World Medical Association Declaration of Helsinki [43]. All patients gave written informed consent before enrollment in the present study. The patients were fully informed with different aspects and side effects related to the medication. Confidentiality and personal privacy were respected at all levels of the study. Periodical assessment of the physical condition of the patients and the occurrence of any side effects were recorded in all stages of the study. 

##### Patient Selection

(a) Inclusion Criteria

Inclusion criteria were: (a) female or male volunteers with clinically diagnosed mild to moderate AGA (patterned hair loss) that was classified according to Hamilton classification system [44] for males and the Ludwig system [45] for females; (b) 19–45 years; (c) able to follow the study directions and (d) capable of attending the follow-up visits for the total study period.

(b) Exclusion Criteria

Patients who (a) had significantly abnormal physical or laboratory evaluation results; (b) suffered from severe medical problems, such as renal and heart diseases; (c) pregnant or lactating women; (d) had a history of hair transplantation; (e) or used other topical or systemic treatment of hair loss within the last six months were excluded.

The sample size was calculated using nMaster 2.0 software (Department of Biostatistics, Christian Medical College, Vellore, India). The nMaster software 2.0 uses STATA, EpiInfo, nQuery, and others for sample size calculation.

A detailed clinical record was prepared on a predesigned pro forma, and all the patients were subjected to detailed history. The volunteers were asked not to change their dietary habits, lifestyle, and hair care habits. Moreover, they were instructed not to take any other topical or systemic medications for the treatment of hair loss. The nature of the disease and prognosis of the treatment modality was explained to the patient before enrolling them. Routine investigations, such as complete blood count, renal and liver function test, random blood sugar, viral markers such as HIV, hepatitis B surface antigen, hepatitis C, and thyroid function test were carried out before commencing the treatment to rule out infection, thyroid disorder, anemia, and systemic illness.

##### Method

Randomization has great importance in clinical studies because it prevents bias in selection and provides comparable tested groups [46]. A total of 80 patients were randomly divided as 1:1 ratio into two groups (group A and group B). Group A (*n* = 40) was treated with the optimized SV-loaded nanospanlastic dispersion. Group B (*n* = 40) was treated with marketed 5% minoxidil lotion. Group A was prescribed an application of 2–3 mL of the optimized SV-loaded SNVS twice daily on the affected areas of the scalp. Group B was prescribed an application of 2–3 mL of 5% minoxidil lotion (Hair Plus Back^®^) twice daily on the affected areas of the scalp. They were explained the side effects, such as initial hair loss for 1–2 months, headache, redness, etc.

Baseline assessment of all the patients was conducted before starting therapy and then monthly until the end of the study after 6 months. Results were evaluated using clinical photographs (iPhone 6, Apple, Cupertino, CA, USA) and dermoscopy (A one tab, Bomtech, Electronics Co, Seoul, Korea). The number of hair follicles, the hair shaft diameter, and hair follicle density were recorded as dermoscopic parameters for evaluation of the hair growth. Moreover, at each visit, physical condition was assessed through medical examination and any side effects were recorded.

#### 2.2.5. HPLC Assay of SV

The quantification of SV was performed using a validated HPLC procedure [47] using the Dionex UltiMate 3000RS HPLC system (Thermo ScientificTM, DionexTM, Sunnyvale, CA, USA). The HPLC system involved an LPG-3400RS quaternary pump, an Inertsil reversed-phase C18 (150 × 4.6 mm × 5 μm) column, a WPS-3000RS autosampler, a TCC-3000RS column thermostat, and a DAD-3000RS diode array detector. The software used for collecting and processing data was Chromeleon 7. For analysis, the mobile phase was a mixture of acetonitrile and 0.32% monobasic potassium phosphate solution (60:40) adjusted to pH 3 using orthophosphoric acid. The injection volume was 20 μL, the flow rate was 1 mL/min. All assays were performed at room temperature.

The method was previously validated by Phaechamud et al. [47]. In addition, we confirmed the validation of this method according to the ICH guideline Q2 (R1) [48] by the determination of linearity, limit of detection (LOD) limit of quantification (LOQ), selectivity, precision and accuracy of the developed method. The validation of the proposed method, according to the ICH guidelines, verified the reasonable applicability of this technique [39]. The calibration curve was found to be linear with a correlation coefficient value of 0.9997. The LOD and LOQ offer an indication of the sensitivity of the validated method [49]. LOD and LOQ were found to be 0.521 and 1.579, respectively. The selectivity of this method was demonstrated by the good separation of SV peak with no interference from the other eluting peaks. Percentage precision was less than 2.0% indicating reasonable repeatability of this technique. The intraday precision and the interday precision were found to be 0.123–0.155% and 0.149–0.179%, respectively. The percentage recovery was in the range of 98.82–99.78%, with a percentage relative standard deviation (RSD) of 0.78–1.69% which displayed the high accuracy of this method.

From the previous results, it is worth mentioning that the developed HPLC technique was sensitive, selective, precise, and accurate. The detection wavelength of SV was 220 nm. HPLC chromatogram of SV is illustrated in Supplementary Data, Figure S1.

#### 2.2.6. Statistical Analysis

Statistical analysis was performed via Student’s *t*-test and one-way ANOVA, using SPSS-11 software (SPSS. Inc., Chicago, IL, USA). Analysis of the results obtained from the 2^3^ factorial design was conducted by ANOVA using Design-Expert software, Version 7.0.0 (Stat-Ease, Inc., Minneapolis, MN, USA) to explore the impact of the chosen independent variables on EE% and Q_12h_. *P*-value < 0.05 denotes statistically significant differences.

## 3. Results

### 3.1. The Preliminary Screening Study 

The preliminary screening test was performed in order to choose the most suitable levels of different independent variables on the basis of achieving the highest EE%. Concerning the ratio of Span 60 to EA, it was noticed that the EE% of SV-loaded SNVs at 50:50 ratio was lower than the other two ratios. That may be attributable to increasing the drug leakage from the SNVs at lower concentrations of Span 60 as a result of increasing the fluidization of the vesicular membrane [21].

With respect to the type of EA, SV-loaded SNVs containing Brij 35 exhibited the lowest EE% compared to those containing Tween 80 and Cremophor RH. That might be explained on the basis of the shorter carbon (C12) chain length of Brij 35 and its higher HLB value (16.9) [50]. Additionally, it is worth noting that the high rotation speed has a negative effect on the drug entrapment into SNVs. The rotation speed of 1500 rpm has the lowest EE%, which may be attributed to decreasing the size of the SNVs upon increasing the rotation speed [51].

According to the previous findings, eight SV-loaded SNVs were fabricated using Tween 80 and Cremophor RH as EAs at 60:40 and 8:20 Span 60 to EA ratios and with rotation speeds of 500 and 1000 rpm.

### 3.2. Analysis of Factorial Design

The factorial design was used for analyzing the influence of different variables on the characteristics of the fabricated formulations [52]. The composition and the measured responses (EE% and Q_12h_) of SV nanospanlastics are exhibited in Table 1. Regression equations were established by Design-Expert^®^ Software to illustrate the relationship between the selected independent variables and the measured responses and to identify the relative influence of various factors by comparing the factor coefficients. The positive sign in front of the factor coefficients revealed a positive effect on the measured responses while the negative sign exhibited a negative impact on the measured responses.

Table 2 investigates the output data of the 2^3^ factorial design of SV-loaded nano-spanlastics. Data of both responses (Y1 and Y2) exhibited significant fit to the linear model because their R^2^ values were relatively high (R^2^ = 0.9731 and 0.9512, respectively). Therefore, the obtained equations are considered to be statistically valid and form an acceptable fit to the obtained data [53]. The predicted R^2^ values measured the response value predictability of the present model and offer an insight into how good the model could fit the new results given by the same relationship that was modeled. The adjusted R^2^ is the modified form of R^2^ that investigates how good the present model would fit the observed results. Therefore, the predicted and adjusted R^2^ should be close to each other. If they are not, there might be a problem with either the model or the data. In the present model, the difference between the predicted and the adjusted R^2^ of Y1 and Y2 values is less than 0.2. Therefore, they were in reasonable harmony [16]. In addition, adequate precision could estimate the signal to noise ratio in order to make sure that the present model is suitable for navigating the design space. Adequate precision was found to be higher than the desired value (5) for Y1 and Y2. It determines the signal to noise ratio and shows that this model could be used for navigating the design space.

Regression equations in terms of coded factors

(4) EE (Y1)=+86.03+8.18× X1+2.17× X2 −2.70× X3

(5) Q12h (Y2)=+75.48+3.45×X1+5.15×X2+4.66× X3

ANOVA analysis was used for evaluating the significance of different factors (Table 3). The model terms are considered to be significant when *P*-value is less than 0.05 [54].

#### 3.2.1. The Effect of Formulation Variables on EE% of SV-Loaded SNVs

**Figure 1:**
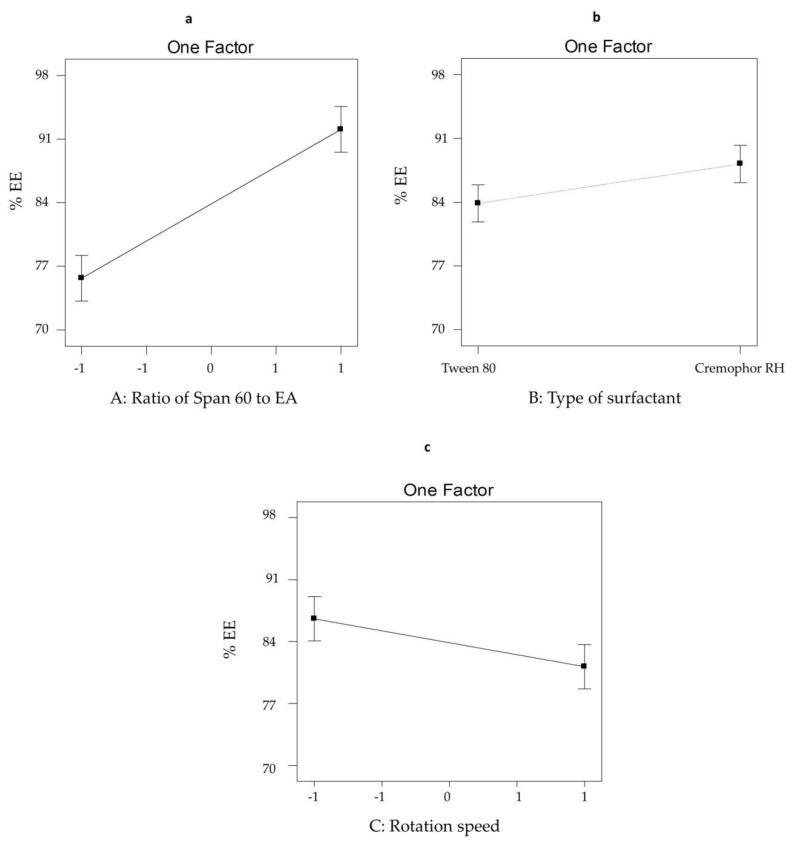
The influence of different independent variables (**a**) ratio of Span 60 to EA, (**b**) type of surfactant and (**c**) rotation speed on %EE of SV-loaded nanospanlastics according to 2^3^ factorial design. Abbreviations: SV, Sodium valproate; EE, entrapment efficiency.

The ability of nanospanlastic vesicles to successfully entrap significant drug amount is considered an important parameter for optimum dermal delivery. Table 1 investigates that the percentage of SV entrapped within different spanlastic formulations ranged from 70.18 ± 0.62 to 97.68 ± 1.93%. The influence of different formulation variables on the %EE of SV-loaded nanospanlastics is demonstrated in Figure 1. The ANOVA results (Table 3) showed that all the studied factors have a significant impact on the %EE of the fabricated nanospanlastic formulations.

With respect to the effect of the ratio of Span 60: EA (X1) on EE%, it was obvious that X1 has a positive effect (*p* < 0.001) on drug entrapment in the nanospanlastic vesicles. This might be due to decreasing the fluidization of the vesicular membrane at high concentrations of Span 60 [21].

In addition, the type of EA (X2) had a significant effect on %EE (*p* < 0.05). Tween 80-based nanospanlastic vesicles showed lower EE% than Cremophor RH-based vesicles. That might be due to the more steric structure of Tween 80. Therefore, increasing its concentration may result in more drug leakage because no more area on the surface of the nanovesicles to be occupied. Whereas, Cremophor has a non-branched saturated chain that permits higher drug entrapment within the nanospanlastic vesicles [55]. These results are in agreement with other studies like Petchsomrit et al. [56].

Concerning the rotation speed (X3), it was found that X3 had a negative influence (*p* < 0.05) on %EE of SV nanospanlastic vesicles. That could be attributed to decreasing the size of SV-loaded nanovesicles on increasing the rotation speed [51].

#### 3.2.2. The Effect of Formulation Variables on Q_12h_ from SV-Loaded SNVs

It is important to ensure sink conditions for performing the in vitro release studies. Sink conditions are attained when the volume of the dissolution medium is at least three times the volume required to obtain a saturated drug solution. The solubility of SV in phosphate buffer (pH = 7.4) was 2.71 ± 0.56 mg/mL.

**Figure 2:**
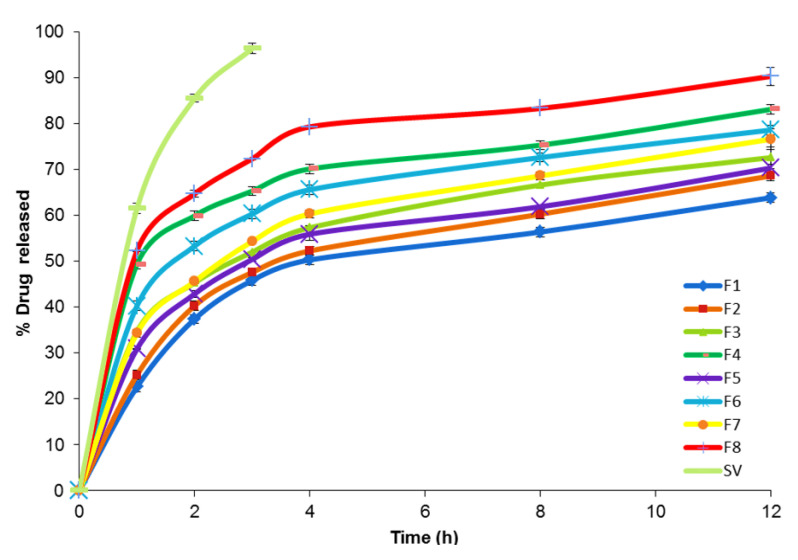
In vitro release profile of SV-loaded nanospanlastic vesicles through semi-permeable cellulose membrane for 12 h, (*n* = 3). Abbreviations: SV, Sodium valproate.

Figure 2 demonstrates the results of the in vitro release study of different SV-loaded nanospanlastic vesicles. Q_12h_ varied from 63.84 ± 1.75% to 90.27 ± 1.98%, Table 1. However, SV dispersion exhibited a higher drug release of 96.33 ± 1.02% after 3 h. That could be attributable to the prolonged release of SV from the nanospanlastic vesicles. In addition, a higher percentage in vitro release profile of free drug investigates that the sink condition was successfully accomplished and the in vitro release profile of SV was not impeded by the semipermeable cellulose membrane [31].

**Figure 3:**
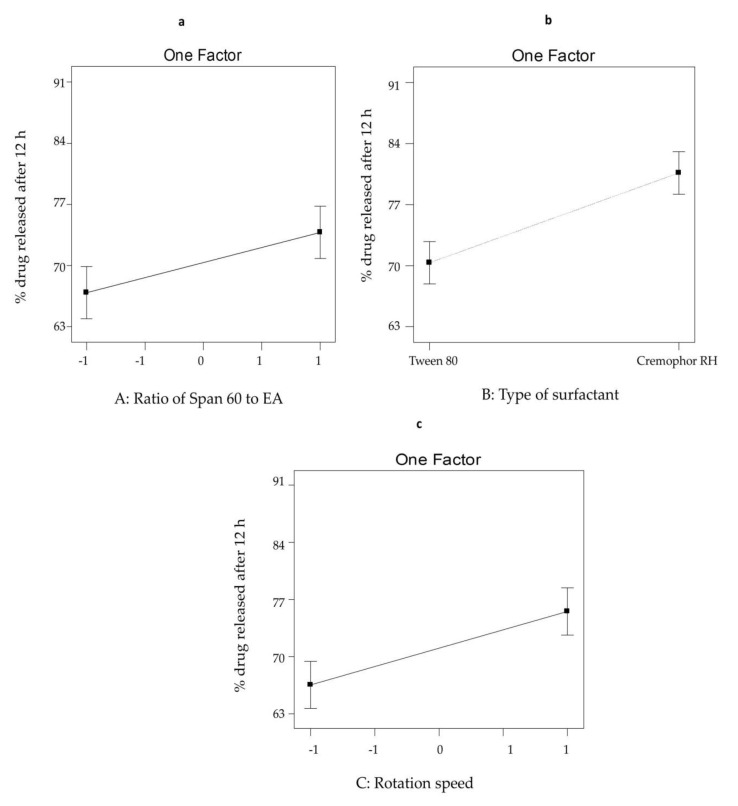
The influence of different independent variables (**a**) ratio of Span 60 to EA, (**b**) type of surfactant and (**c**) rotation speed on Q_12h_ of SV-loaded nanospanlastics according to 2^3^ factorial design. Abbreviations: SV, Sodium valproate; Q_12h_, % drug released after 12 h.

The influence of different independent variables on Q_12h_ of SV-loaded nanospanlastic vesicles is illustrated in Figure 3. ANOVA analysis (Table 3) showed that the ratio of Span 60: EA (X1) showed a significant positive impact on Q_12h_ of SV-loaded nanospanlastic vesicles (*p* < 0.05). That could be explained on the fact that as the amount of EA increased, mixed micelles are formed that are considered to be less sensitive to the concentration gradient than the spanlastic vesicles leading to reduced drug release [15]. With respect to the type of EA (X2), it was noticed that X2 had a significant effect on Q_12h_ (*p* < 0.01). Cremophor RH-based spanlastics exhibited higher Q_12h_ than Tween 80-based spanlastics. That may be explained on the basis of the high solubilizing capacity of Cremophor RH [56,57,58]. Moreover, the rotation speed (X3) had a significant positive impact on Q_12h_ (*p* < 0.01) that may be due to decreasing the size of nanospanlastic vesicles [59] and consequently increasing drug leakage. These findings are also comparable with the results of EE %.

**Figure 4:**
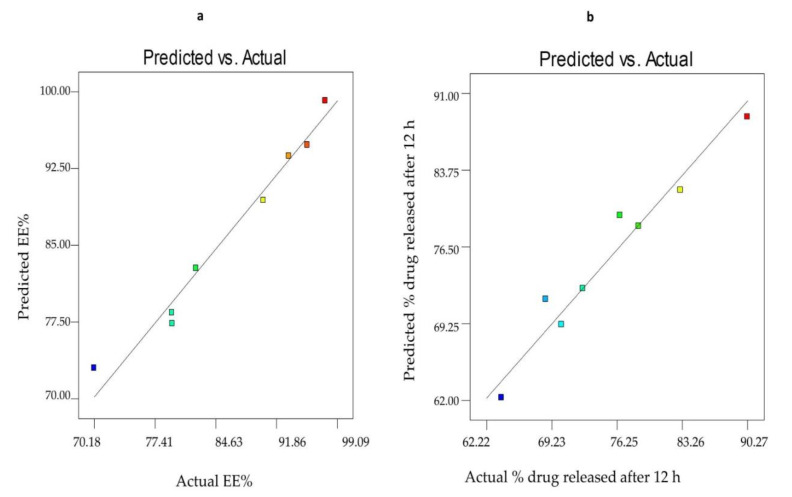
Linearity plots of SV-loaded SNVs shown as observed versus predicted values for (**a**) % EE and (**b**) Q_12h_. Abbreviations: SV, Sodium valproate; SNVs, spanlastic nanovesicles; EE, entrapment efficiency; Q_12h_, % drug released after 12 h.

Figure 4 investigated the linearity plots between the observed and the predicted values of both EE % (Y1) and Q_12h_ (Y2) of SV-loaded nanospanlastics. It is clear that there is an excellent fit between the observed and predicted values of both responses in the present model.

#### 3.2.3. Optimization of SV-Loaded Nanospanlastics

The optimization process is generally intended for the implementation of systematic strategies to attain the best possible process combinations from which a high-quality pharmaceutical formulation could be produced. It involves studying the impact of different independent variables on the characteristics of pharmaceutical formulations with subsequent choice of the best performing formula. The optimum values of different variables were determined through numerical analysis using the Design-Expert software program for selecting the optimized SV-loaded nanospanlastic formula [60].

The performance of pharmaceutical products is commonly described by a number of response variables. Simultaneous optimization of these variables is required for selecting the optimized formula. In the approach of desirability criteria, each response is converted into a desirability value. The total desirability value is the geometric average of different desirability values. The desirability value will be zero if the response value is beyond the acceptable range. It would be one when the response value is on target. It would lie between zero and one if the response value lies within the tolerance range but not on the target. The desirability value becomes closer to one when the response value approaches the target [61].

**Figure 5:**
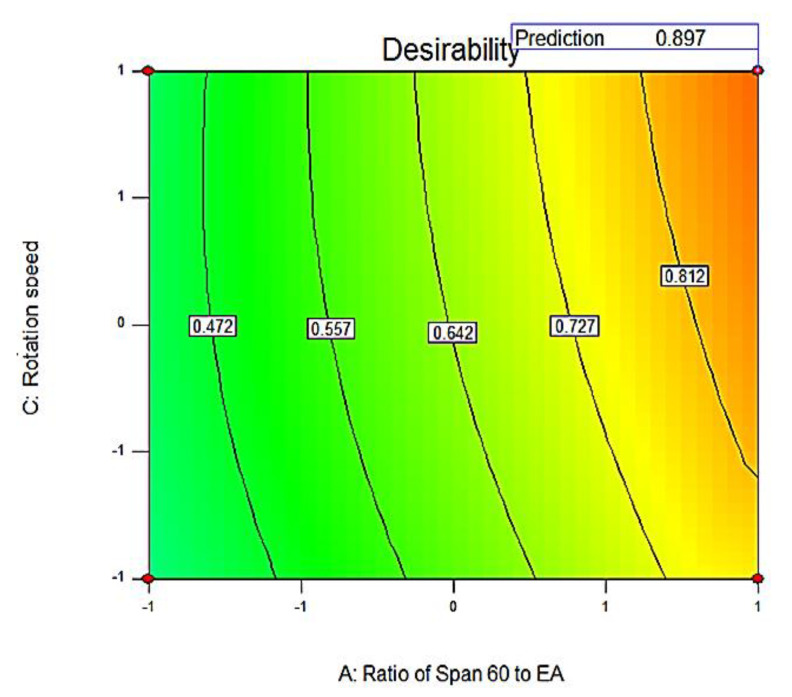
Contour plot showing the overall desirability of the optimized SV-loaded nanospanlastic formula (F8) as a function of independent variables. Abbreviations: SV, Sodium valproate.

In the present design, different formulations were optimized for the EE % (Y1) and Q_12h_ (Y2) on the basis of maximizing both EE % and Q_12h_. Results showed that F8 had the highest desirability value (0.897) (Figure 5). Moreover, the predicted values of F8 were 93.68% and 88.74% for Y1 and Y2, respectively. The percentage relative error was found to be less than 5 (0.36 and −3.26 for Y1 and Y2, respectively), investigating the fitness of the present model [31]. Hence, it could be considered as the optimized nanospanlastic formula that is selected for further investigation.

### 3.3. Characterization of the Optimized SV-Loaded SNVs

#### 3.3.1. Morphological Characterization by SEM

**Figure 6:**
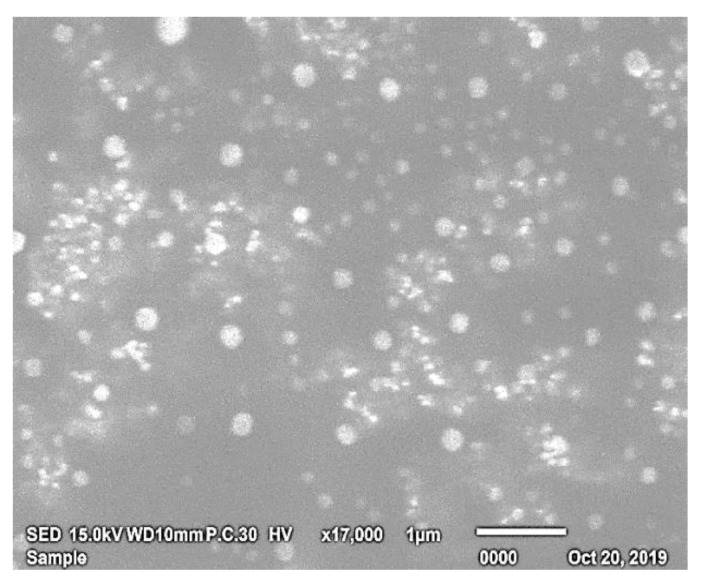
Scanning electron micrograph of the optimized SV-loaded nanospanlastic formula (F8). Abbreviations: SV, Sodium valproate.

The optimized SV-loaded nanospanlastic vesicles (F8) appeared as a well-identified homogenous dispersion of non-aggregating spherical vesicles (Figure 6). The spherical shape of SV-loaded SNVs may be explained on the basis of the amphipathic nature of the non-ionic surfactants that results in a spontaneous formation of bilayers in aqueous medium and their tendency to minimize the surface free energy [33,62].

#### 3.3.2. Fourier Transform Infrared (FTIR) Spectroscopy

**Figure 7:**
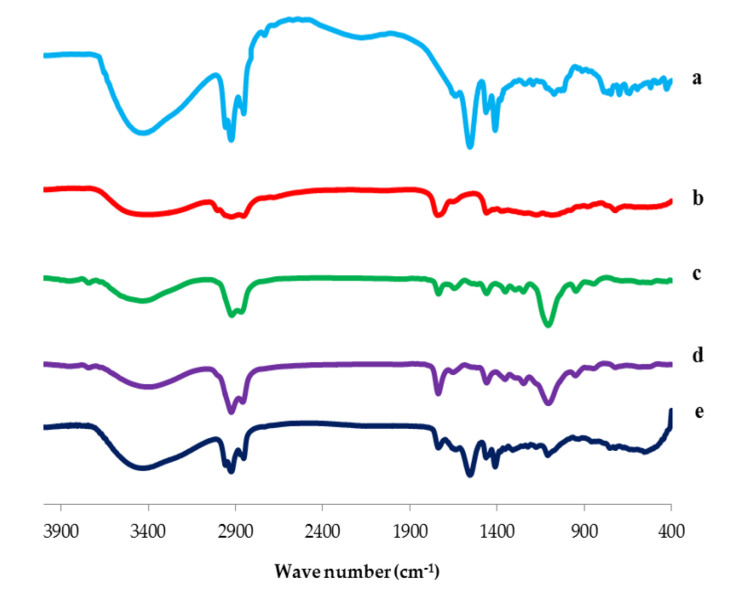
FTIR spectrum of (**a**) SV, (**b**) Span 60, (**c**) Cremophor RH, (**d**) Plain spanlastics and (**e**) optimized SV-loaded SNVs (F8). Abbreviations: SV, Sodium valproate.

The FTIR spectra of SV, Cremophor RH 40, Span 60, plain (drug-free) nanospanlastic formula and the optimized formula (F8) are illustrated in Figure 7. The FTIR spectrum of SV exhibited characteristic vibrations in the region of 2937–2848 cm^−1^ associated with aliphatic C-H stretching. The vibrational peaks detected at 1544–1404 cm^−1^ were attributed to COO- stretching, respectively [63,64].

The IR spectrum of Cremophor RH exhibited a broad band at 3386 cm^−1^ associated with OH group; bi-forked peak at (2904–2856 cm^−1^) is assigned to the aliphatic –CH; band at 1725 cm^−1^ originates from the carbonyl group of ester and a strong broad band at 1093 cm^−1^ corresponding to the C–O–C stretching vibration [65]. Span 60 exhibited characteristic band assignments, such as aliphatic O–H stretch (3410 cm^−1^), C–H stretch (2936 cm^−1^) and C=O stretch of ester (1745 cm^−1^) [66].

The FTIR spectrum of the plain spanlastic formula exhibited the characteristic peaks of both Span 60 and Cremophor RH. Reducing the intensity of both Cremophor RH and Span 60 peaks could be interpreted on the basis of lipid bilayer formation in the plain spanlastic vesicles [33].

Moreover, the FTIR spectrum of the optimized spanlastic formula (F8) displayed the characteristic peaks of SV and different excipients that exhibited the absence of interactions between SV and different excipients. The reduced intensity and minor shifting of the characteristic peaks of SV could be attributed to the presence of various bonds between SV and other excipients, such as hydrogen bond, dipole interactions, or Van der Wall forces. These bonds lead to higher entrapment of SV in the nanospanlastic vesicles [33].

#### 3.3.3. Differential Scanning Calorimetry (DSC) Study

**Figure 8:**
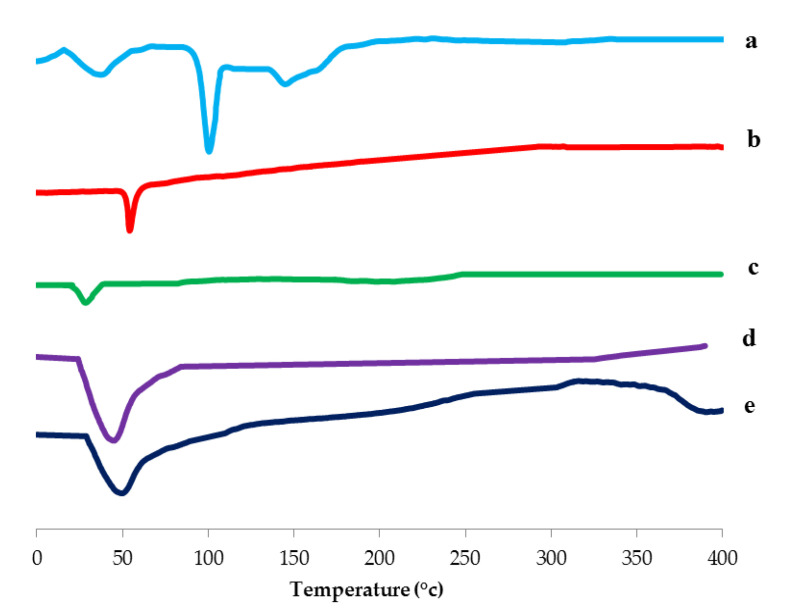
DSC thermogram of (**a**) SV, (**b**) Span 60, (**c**) Cremophor RH, (**d**) Plain spanlastics and (**e**) optimized SV-loaded SNVs(F8). Abbreviations: SV, Sodium valproate.

DSC thermograms of SV, Span 60, Cremophor RH, plain spanlastics and the optimized spanlastic formula are illustrated in Figure 8. The DSC thermogram of SV showed a characteristic endothermic peak at 99.3 °C which revealed the crystallinity of SV [63,64,67]. The DSC thermogram of Span 60 exhibited an endothermic peak at 54.4 °C that corresponds to its transition temperature [68]. Cremophor RH showed an endothermic peak at 26.1 °C attributed to its melting transition [65,69]. The plain (drug-free) nanospanlastic formula exhibited the appearance of a new endothermic peak at 40.2 °C that may be attributable to the interaction between the components of the nanospanlastic vesicles during the development of the lipid bilayer [66]. F8 exhibited the disappearance of the endothermic peak of SV and a shift of the endothermic peak of the lipid bilayer to 45.6 °C. That may be explained on the basis of good encapsulation of SV within the nanospanlastic vesicles. Moreover, the presence of SV in the amorphous state in the nanospanlastic dispersion increases the phase transition temperature of the nanospanlastic vesicles. These results are in agreement with data reported by Kakkar and Kaur [70].

#### 3.3.4. Determination of Particle Size and Zeta Potential

**Figure 9:**
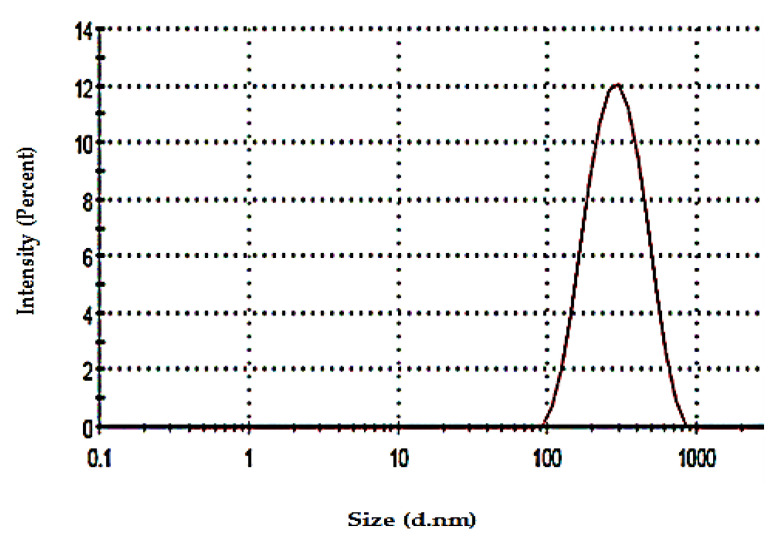
Particle size distribution curve of the optimized SV-loaded SNVs (F8). Abbreviations: SV, Sodium valproate.

The vesicular size of the optimized SV-loaded spanlastic formula (F8) was found to be 222.9 ± 2.39 nm, with a PDI value of 0.34. The small value of PDI, less than 0.5, reflects the homogenous size distribution of the nanospanlastic vesicles, Figure 9.

Zeta potential is a measure of the net charge of the nanovesicles and is considered as an indirect description of the stability of colloidal dispersion. The colloidal dispersion that has large positive or negative zeta potential is considered to be stable. The high charge on the vesicle surface creates repulsion between the vesicles that makes them stable and preserves them from agglomeration providing a uniformly distributed suspension [71]. The optimized spanlastic formula (F8) has a high negative zeta potential value (−35.63 mv), indicating that the chosen formula is stable. The negative zeta potential values of spanlastic dispersions, containing non-ionic surfactants, were also reported by other researchers including Fahmy et al. [21], Al-mahallawi et al. [35], Farghaly et al. [14] and Basha et al. [72]. The reason for the negative zeta potential values of nanovesicles, containing non-ionic surfactants, was discussed by many researchers. Caracciolo et al. [73] investigated that a negative zeta potential is produced in niosomal vesicles containing Tween 20 and cholesterol, even in the absence of charge-inducing agents, owing to the orientation of Tween 20 and cholesterol hydroxyl groups with respect to the aqueous environment and the subsequent redirection of the ionic charges in water. Junyaprasert et al. [74] reported that the Span 60-based blank niosomes demonstrated a negative value of the zeta potential which could be attributable to the adsorption of counterions at the vesicle surface. Additionally, Essa et al. [75] found that Span 40-based control niosomes exhibited a negative zeta potential value that may be explained on the basis of the preferential adsorption of hydroxyl ions on the surface of niosomal vesicles. Other researchers, such as Pawar et al. [76], have demonstrated that the negative surface charge of Span 60-based niosomes of Bifonazole might be attributed to the ionization of free groups present on the niosomal surface.

The cell membrane is negatively charged. However, the high cellular uptake of the negatively charged nanoparticles without any repulsion could be attributable to the non-specific adsorption of nanoparticles on the cellular membrane and the development of clusters of the nanoparticle [77].

#### 3.3.5. Measurement of Vesicle Elasticity

The deformability of the nanospanlastic vesicles denotes their ability to squeeze through narrow pores of the biological membranes that are smaller than their own diameter without rupture. This character is an important criterion that distinguishes them from conventional niosomal vesicles. The DI of the optimized SV-loaded SNVs (22.57 ± 1.5) was significantly (*p* < 0.001) higher than that (0.98 ± 0.03) of the corresponding niosomal dispersion. That could be attributed to the addition of EAs that improve the elasticity of SNVs by fluidization of the lipid bilayer [16].

The vesicle size of both formulations was determined before and after extrusion (Supplementary Data, Table S2). It was noted that there was a significant decrease (*p* < 0.001) in the vesicle size of the niosomal formula, after extrusion. That could be attributable to the absence of elasticity of the niosomal vesicles, which results in their rupture during passage through the membrane [78]. However, SV-loaded SNVs could retain their vesicular size with no significant difference (*p* > 0.05) in the size after extrusion. That could be explained on the basis of the elasticity and deformability of the SNVs after the addition of EA [79,80].

#### 3.3.6. Comparative ex vivo Permeation between the Optimized SV-Loaded SNVs and Free Drug

The ex vivo permeation of the optimized SV-loaded SNVs through rat skin was performed to evaluate the impact of the nanospanlastic vesicles on improving the delivery of SV through the skin. The ex vivo permeation studies, using the diffusion technique, give a possibility for fast and initial screening of drug permeability for the optimization of different products [81]. Using excised human skin for evaluating the dermal drug delivery is restricted by several factors, such as laboratory facilities and ethical considerations. Besides, a great difference has been found in the permeability between the skin specimens taken from different donors (inter-individual difference) or even the same donor (intra-individual variation).

The skin of rodent animals (rats, mice and pigs) are the most commonly used specimens in the ex vivo skin permeation studies because of their low cost, availability, easy handling and relatively small size [82].

Among rodents, the rat skin is the most widely used rodent model due to its structural similarity to human skin [83]. Many studies have been performed in order to compare the ex vivo permeation through rat and human skins. They found that there was a reasonable correlation in the drug permeation between the rat and human skins [84,85]. Consequently, the rat skin could be used for calculating the skin permeation parameters, such as flux, permeability coefficient and diffusion coefficient of topically-applied drugs. Besides, Takeuchi et al. [86] concluded that the inter-individual variation in the drug permeation through the rat skin was significantly lower than that through the human skin, and the permeation rates for rat and human skins correlated well with each other. Hence, rat skin is often used as an alternative to human skin in the ex vivo permeation studies.

Regarding pig (porcine) skin, Abd et al. [87] demonstrated that pig skin is histologically similar to human skin. However, the SC of both species differs markedly. The lipids of human SC were packed predominantly in an orthorhombic lattice, while porcine SC lipids are mainly arranged in a less dense hexagonal lattice. Moreover, the compositions of SC lipid (free fatty acid and ceramide) in the two species are different. Hence, the ex vivo permeation through both species would be different because the SC has an important role in the drug permeability. The orthorhombic packing arrangement is present in human, rat and mice skins [83]. The skin of mice performed similarly to the human neck, cheek and inguinal skin in the ex vivo skin permeation [88].

**Figure 10:**
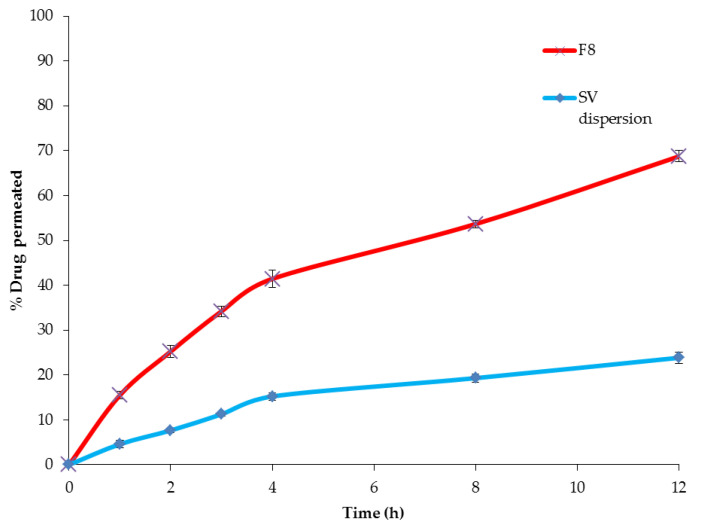
Ex vivo permeation profile of SV aqueous dispersion and the optimized SV-loaded SNVs (F8) through rat skin for 12 h, (*n* = 3). Abbreviations: SV, Sodium valproate; SNVs, spanlastic nanovesicles.

Based on the permeation profiles (Figure 10), it is worthy to mention that the encapsulation of SV into the nanospanlastic vesicles resulted in a significant improvement (*p* < 0.001) in the ex vivo permeation of SV relative to the aqueous drug dispersion. The permeation of SV aqueous dispersion was 23.85 ± 1.49% after 12 h, whereas the optimized SV-loaded SNVs exhibited higher ex vivo permeation reaching 68.73 ± 1.65% after 12 h. Furthermore, the ex vivo permeation parameters (Table 4) revealed that the optimized nanospanlastic formula (F8) had a significant enhancement of SV flux across the skin (*p* < 0.05) relative to SV dispersion, with an enhancement ratio of 3.00. The enhanced permeation of SV-loaded SNVs could be attributable to the deformability and elasticity of the nanospanlastic vesicles that facilitate permeation through biological membranes achieving higher permeation relative to the free drug [89]. Besides, these nanospanlastic vesicles could serve as a penetration enhancer that could overcome the barrier properties of SC [90].

### 3.4. Comparative Study between the Optimized SV-Loaded SVs and Marketed Minoxidil Lotion by Dermoscopic Evaluation for Treatment of AGA

A comparative clinical study was performed to compare both the efficacy and the safety of SV-loaded SNVs versus topical minoxidil in the treatment of AGA using dermoscopic evaluation.

#### 3.4.1. Distribution of Cases in the Study Groups

The study included 80 patients with AGA who were classified into two groups each of 40 patients according to treatment regimen; SV-loaded SNVs treated group (group A) and minoxidil treated group (group B). Table 5 demonstrates the demographic distribution of cases in the study groups according to age, sex and occupation. It is obvious that there was no statistically significant difference between the two groups (*p* > 0.05).

In addition, Table 6 investigates the disease criteria among the studied groups. No significant difference (*p* > 0.05) was detected between the two groups with respect to the duration of alopecia, family history of psoriasis and previous treatment (herbal products and vitamins that were used for treatment of hair loss more than 6 months ago).

With respect to the disease classification, the cases were classified according to Ludwig [45] and Hamilton classifications [44] and no statistically significant difference (*p* > 0.05) was detected between the two groups, Table 7.

#### 3.4.2. Result Analysis Using Dermoscopic Parameters

**Figure 11:**
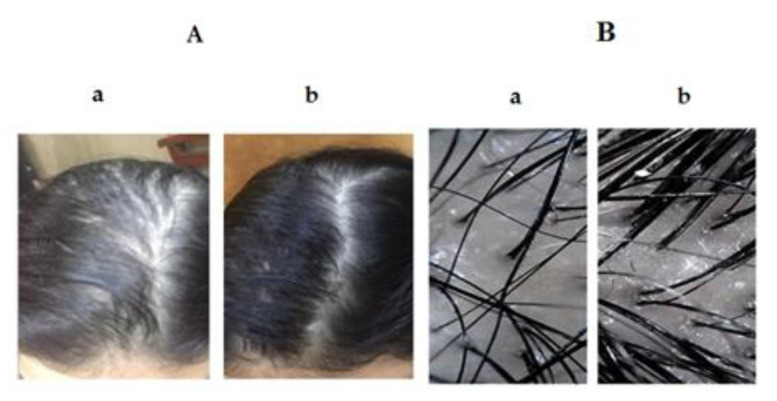
Clinical photographs (**A**), dermoscopic pictures (**B**) of group A; pre-treatment (**a**) and Post-treatment (**b**) with SV-loaded SNVs. Abbreviations: SV, Sodium valproate; SNVs, spanlastic nanovesicles.

**Figure 12:**
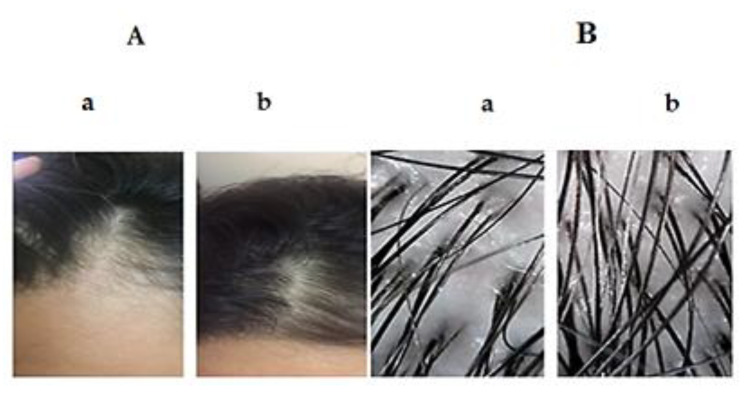
Clinical photographs (**A**), dermoscopic pictures (**B**) of group B; pre-treatment (**a**) and Post-treatment (**b**) with minoxidil.

There are many methods used for the clinical evaluation of patients who suffer from hair loss. These methods could be categorized as non-invasive (dermoscopy), semi-invasive (trichogram), or invasive (scalp biopsy). A valuable insight into the patient diagnosis and treatment could be attained when the results of these techniques are interpreted with a comprehensive clinical picture. More recent studies reported that the use of dermoscopy of scalp and hair in the clinical assessment of hair disorders could provide a higher diagnostic capability than simple clinical examination [91,92,93]. Figure 11 and Figure 12 illustrate the clinical photographs and the dermoscopic pictures of group A and group B, respectively. The dermoscopic examination of scalp and hair permits to visualize both the scalp and hair shaft with high accuracy and appropriate magnification.

**Figure 13:**
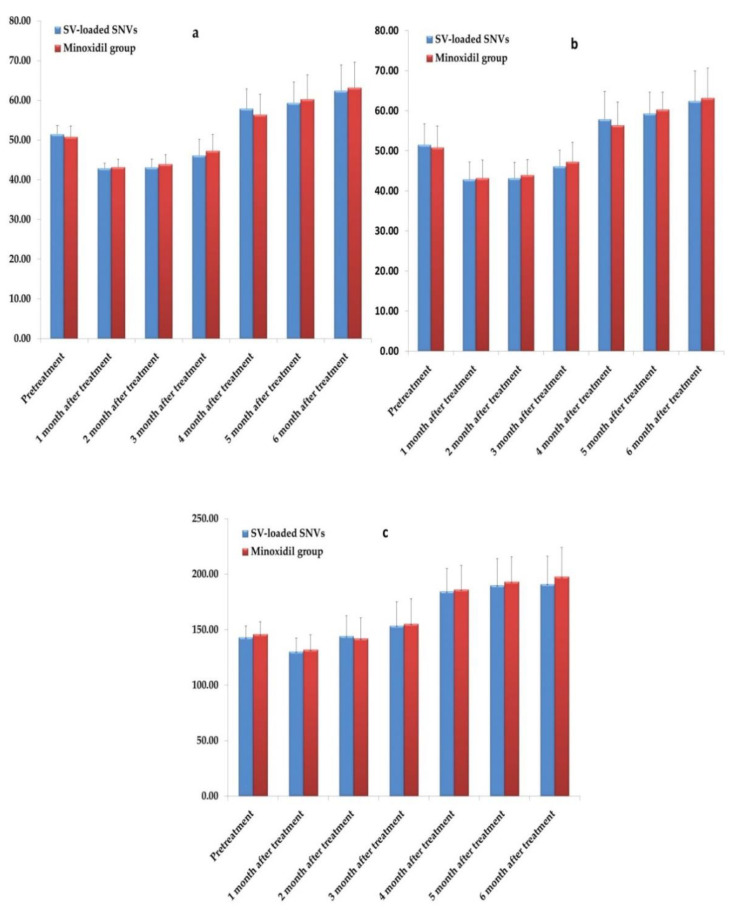
Follow up of (**a**) the number of hair follicles, (**b**) the hair shaft diameter, and (**c**) the hair follicle density for SV-loaded SNVs (*n* = 40) and minoxidil (*n* = 40) groups. Abbreviations: SV, Sodium valproate; SNVs, spanlastic nanovesicles.

The results of both treatments were analyzed at different time points (0, 1st, 2nd, 3rd^,^ 4th, 5th and 6th) months. Before treatment, the mean number of hair follicles was 17.9 ± 2.1/cm^2^ and 18.3 ± 2.75 cm^2^ in group A and group B, respectively, with no significant difference between the two groups (*p* > 0.05). There was an initial decrease in the number of hair follicles at 1st and 2nd months that was more evident in group B with a statistically significant difference (*p* < 0.05) between the two groups (Supplementary Data, Table S3). That may be attributed to the shedding of previous telogen hairs due to the premature entry of the resting hair follicles into the anagen phase of hair growth [94]. However, there was an increase in the number of hair follicles among both groups from the baseline during the 3rd, 4th, 5th and 6th months of the study. The increase was comparable in both groups with no significant difference (*p* > 0.05). After 6 months of treatment, there was a high statistically significant increase (*p* < 0.001) in the mean number of hair follicles to 31.58 ± 6.49/cm^2^ and 32.54 ± 6.39/cm^2^ in group A and group B, respectively. The follow up of the number of the follicles in the cases of both groups is illustrated in Figure 13a.

The decrease in hair shaft diameter is a critical diagnostic criterion for AGA because it denotes the miniaturized hair follicles [95]. Before treatment, the mean hair shaft diameter was 51.5 ± 5.27 µm and 50.7 ± 5.44 µm in group A and group B, respectively, with no significant difference between the two groups (*p* > 0.05). Afterward, there was an increase in the hair shaft diameter at the 4th, 5th and 6th months of the study with no significant difference between the two groups (*p* > 0.05). After 6 months of treatment, the hair shaft diameter increased to 62.4 ± 7.54 µm and 63.19 ± 7.49 µm in group A and group B, respectively, with a high statistically significant difference (*p* < 0.001) between pre-and post-treatment in both groups (Supplementary Data, Table S4). Figure 13b shows the follow up of the hair shaft diameter in the cases of both groups.

The hair follicle density denotes the density of hair follicle structures present in the scalp. Before treatment, the mean hair follicle density was 143.2 ± 10.11/cm^2^ and 146.04 ± 11.23/cm^2^ in group A and group B, respectively, with no significant difference between the two groups (*p* > 0.05). There was an increase in the hair density with no statistically significant difference (*p* > 0.05) between the two groups at the 3rd, 4th, 5th and 6th months of the study. After 6 months of treatment, there was a high statistically significant increase in the mean hair follicle density (*p* < 0.001) to 191.26 ± 24.72/cm^2^ and 197.71 ± 26.23/cm^2^ in group A and group B, respectively (Supplementary Data, Table S5). The follow up of the hair follicle density in the cases of the study in both groups was illustrated in Figure 13c.

The previous results show a positive response to both treatments with no comparable difference between them and verify the efficacy of SV-loaded SNVs in the treatment of AGA.

Regarding the patient’s satisfaction, there was no statistically significant difference between the two study groups regarding the degree of patient’s satisfaction (*p* > 0.05), Table 8.

**Figure 14:**
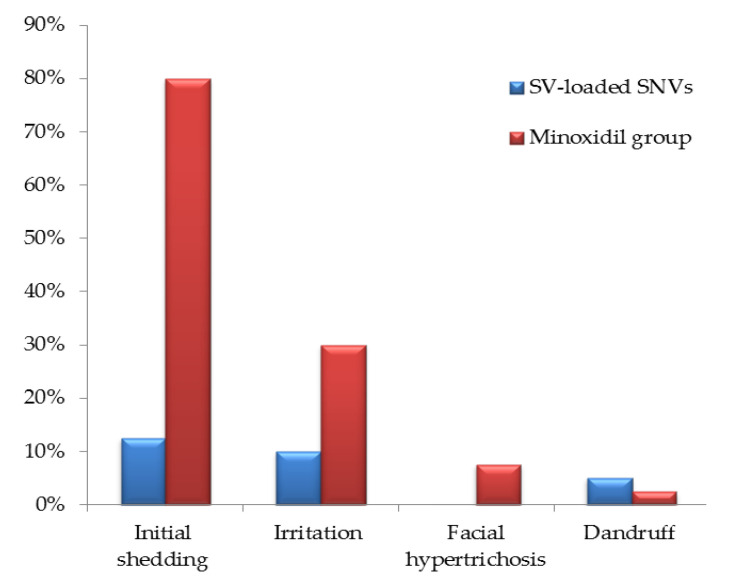
Analysis of the side effects in the cases of the study for SV-loaded SNVs (*n* = 40) and minoxidil (*n* = 40) groups. Abbreviations: SV, Sodium valproate; SNVs, spanlastic nanovesicles.

Figure 14 investigates the associated side effects in both groups. It is clear that the initial shedding and irritation observed in group A were significantly lower than that detected in group B (*p* < 0.001 and *p* < 0.01, respectively). Moreover, 7.5% of cases suffered from facial hypertrichosis in group B, whereas no facial hypertrichosis was detected for group A (Supplementary Data, Table S6). Regarding dandruff, 5% and 2.5% of cases in group A and group B, respectively, suffered from dandruff with no statistically significant difference between the two groups (*p* > 0.05). However, the two cases with dandruff in group A were reported to have it before the onset of treatment for which ketoconazole shampoo was commenced. These results demonstrate the safety of SV-loaded SNVs with respect to minoxidil.

The above results investigate both the efficacy and safety of SV-loaded SNVs in the treatment of AGA, which could improve patient compliance and medication adherence that may be an important step toward enhancing the proper use of medication.

## 4. Conclusions

The current study demonstrated the repurposing of topical SV for the treatment of AGA and the development of SV-loaded nanospanlastics for improving its dermal delivery. Eight SV-loaded nanospanlastic formulations were successfully fabricated, according to 2^3^ factorial design, by the ethanol injection method using Span 60 and EAs. Design-Expert software was used to select the optimized formula according to the desirability criteria on the basis of maximizing both EE % and Q_12h_. The optimized SV-loaded SNVs (F8) appeared as spherical elastic nanovesicles with reasonable EE % of 93.34 ± 0.92% and prolonged Q_12h_ of 90.27 ± 1.98%. F8 exhibited higher ex vivo permeation than free drug due to the higher elasticity of the nanospanlastic vesicles which improved drug permeation through biological membranes. The comparative clinical study exhibited no significant difference, in the therapeutic efficacy, between F8 and marketed minoxidil lotion. Additionally, F8 demonstrates less adverse effects than minoxidil lotion with no occurrence of facial hypertrichosis. In summary, the obtained results suggest that the nanospanlastics of SV can be a promising drug delivery system that improves the topical delivery of SV for safe and effective treatment of AGA.

## Figures and Tables

**Table 1:** Experimental runs, independent variables and measured responses in 2^3^ factorial design for SV-loaded nano-spanlastics.

Formula Code	Variables				
	Independent			Dependent	
	X1	X2	X3	Y1 *	Y2 *
**F1**	−1	−1	−1	79.44 ± 1.15	63.84 ± 1.75
**F2**	−1	−1	1	70.18 ± 0.62	68.59 ± 1.82
**F3**	−1	1	−1	82.30 ± 0.98	72.59 ± 1.12
**F4**	−1	1	1	79.48 ± 1.33	83.09 ± 1.79
**F5**	1	−1	−1	95.53 ± 1.46	70.29 ± 2.14
**F6**	1	−1	1	90.32 ± 2.18	78.59 ± 1.53
**F7**	1	1	−1	97.68 ± 1.93	76.58 ± 1.29
**F8 ^#^**	1	1	1	93.34 ± 0.92	90.27 ± 1.98
**Independent Variables**		**Low (−1)**		**High (+1)**	
X1: Ratio of Span 60 to EA		60:40		80:20	
X2: Type of EA		Tween 80		Cremophor RH	
X3: Rotation speed (rpm)		500		1000	

Notes: Y1: EE(%), Y2: Q_12h_ (%),* the values are expressed as mean ± SD; *n* = 3, # Optimized Formulation, all formulations contained 8.3% SV. Abbreviations: EE, entrapment efficiency; Q_12h_, drug released after 12 h; EA, edge activator.

**Table 2:** Output data of the 2^3^ factorial design of SV-loaded nano-spanlastics.

Responses	*R^2^*	Adjusted *R^2^*	Predicted *R^2^*	Adequate Precision
EE% (Y1)	0.9731	0.9529	0.8924	17.669
Q_12h_ (Y2)	0.9512	0.9146	0.8047	15.095

**Table 3:** ANOVA for the 2^3^ factorial design of SV-loaded nano-spanlastics.

Dependent Variable	Source	Sum of Squares	df	Mean Square	*F*-Value	*p*-Value
EE% (Y1)	Model	631.81	3	210.65	48.23	0.0013
	X1	535.79	1	535.79	122.71	0.0004
	X2	37.54	1	37.54	8.60	0.0427
	X3	58.45	1	58.48	13.39	0.0216
Q_12h_ (Y2)	Model	481.10	3	160.37	25.98	0.0044
	X1	95.36	1	95.36	15.45	0.0171
	X2	212.39	1	212.39	34.41	0.0042
	X3	173.35	1	173.35	28.08	0.0061

Notes: X1: Ratio of Span 60 to EA, X2: Type of EA, X3: Rotation speed (rpm), *p*-value less than 0.05 indicates model terms are significant. Abbreviations: EE, entrapment efficiency; Q_12h,_ drug released after 12h.

**Table 4:** Ex vivo permeation parameters of the optimized SV-loaded SNVs and SV aqueous dispersion.

Formula	* Jss (µg cm^−2^ h^−1^)	* K_P_ (cm h^−1^)	ER
SV dispersion	2.77 ± 1.16	0.0069 ± 0.65	-------
F8	8.33 ± 1.28	0.0208 ± 1.15	3.00

Notes: F8: The optimized SV-loaded SNVs, * each value represents mean ± SD (*n* = 3). Abbreviations: J_ss_, Steady state flux; K_P_, Permeability coefficient; ER, Enhancement ratio.

**Table 5:** Demographic data among the studied groups.

Demographic Data	SV-Loaded SNVs (*n* = 40)	Minoxidil (*n* = 40)	*p*-Value
**Age/Years**			
Mean ± SD Min-Max	32.32 ± 4.47 (24–41)	34.27 ± 5.64 (25–44)	0.125
**Sex**			
Male	14 (35.0%)	18 (45.0%)	0.161
Female	26 (65.0%)	22 (55.0%)	
**Occupations**			
Worker	25 (62.5%)	22 (55%)	0.226
not working/housewife	15 (37.5%)	18 (45%)	

Notes: *p*-value less than 0.05 indicate model terms are significant. Abbreviations: SV, sodium valproate; SNVs, spanlastic nanovesicles; *P*-value, probability value; Min, minimum; Max, maximum.

**Table 6:** Comparison of the disease criteria among the studied groups.

Disease Criteria	SV-Loaded SNVs (*n* = 40)	Minoxidil (*n* = 40)	*p*-Value
**Disease Duration/Years**			
Mean ± SD Min-Max	7.33 ± 2.05 (2–17)	6.25 ± 2.64 (1–16)	0.118
**Family History of Psoriasis**			
Positive	25 (62.5%)	23 (57.5%)	0.361
Negative	15 (37.5%)	17 (42.5%)	
**Previous Treatment**			
Positive	29 (72.5%)	26 (65%)	0.212
Negative	11 (27.5%)	14 (35%)	

Notes: *P*-value less than 0.05 indicate model terms are significant. Abbreviations: SV, sodium valproate; SNVs, spanlastic nanovesicles; *p*-value, probability value; Min, minimum; Max, maximum.

**Table 7:** Disease classification among the studied groups.

Classification of the Disease	SV-Loaded SNVs (*n* = 40)	Minoxidil (*n* = 40)	*p*-Value
**Female Cases** **(Ludwig Classification)**	(*n* = 26)	(*n* = 22)	
Ludwig I	9 (34.6%)	7 (31.8%)	0.361
Ludwig II	10 (38.5%)	9 (40.9%)	
Ludwig III	7 (26.9%)	6 (27.3)	
**Male Cases** **(Hamilton Classification)**	(*n* = 14)	(*n* = 18)	
Hamilton II	5 (35.7%)	7(38.9%)	0.212
Hamilton III	7 (50%)	8 (44.4%)	
Hamilton IV	2 (14.3%)	3 (16.7%)	

**Notes:***p*-value less than 0.05 indicate model terms are significant. Abbreviations: SV, sodium valproate; SNVs, spanlastic nanovesicles; *p*-value, probability value.

**Table 8:** Patient satisfaction in the studied groups at the end of the study.

Patients Satisfaction	SV-Loaded SNVs (*n* = 40)	Minoxidil (*n* = 40)	*p*-Value
Poor	6 (15%)	7 (17.5%)	0.149
Fair	14 (35%)	13 (32.5%)	
Good	12 (30%)	15 (37.5%)	
Excellent	8 (20%)	5 (12.5%)	

**Notes:***p*-value less than 0.05 indicate model terms are significant. Abbreviations: SV, sodium valproate; SNVs, spanlastic nanovesicles; *p*-value, probability value.

## Supplementary Materials

The following are available online at https://www.mdpi.com/1999-4923/12/9/866/s1, Table S1: Prescreening study for formulation of SV-loaded SNVs, Table S2: Determination of the elasticity of SV-loaded SNVs and the corresponding niosomes, Table S3: The correlation between the hair follicles number/cm^2^ in the studied groups, Table S4: The correlation between the hair shaft diameter (µm) in the studied groups, Table S5: The correlation between the hair density/cm^2^ in the studied groups, Table S6: Side effects among the studied groups, Figure S1: HPLC chromatogram of SV.
