# Supplementary material for: Formulation of Sodium Valproate Nanospanlastics as a Promising Approach for Drug Repurposing in the Treatment of Androgenic Alopecia
Source: Pharmaceutics. 2020 Sep 11;12(9):866. doi: 10.3390/pharmaceutics12090866 (PMC7559423; doi:10.3390/pharmaceutics12090866)
Supplement: Supplementary file 1 [file pharmaceutics-12-00866-s001.pdf]

pharmaceutics

Supplementary Materials: Formulation of Sodium Valproate Nanospanlastics as a Promising Approach for Drug Repurposing in the Treatment of Androgenic Alopecia

F. A. Badria *, Hassan A. Fayed, Amira K. Ibraheem, Ahmed F. State and Eman A. Mazyed

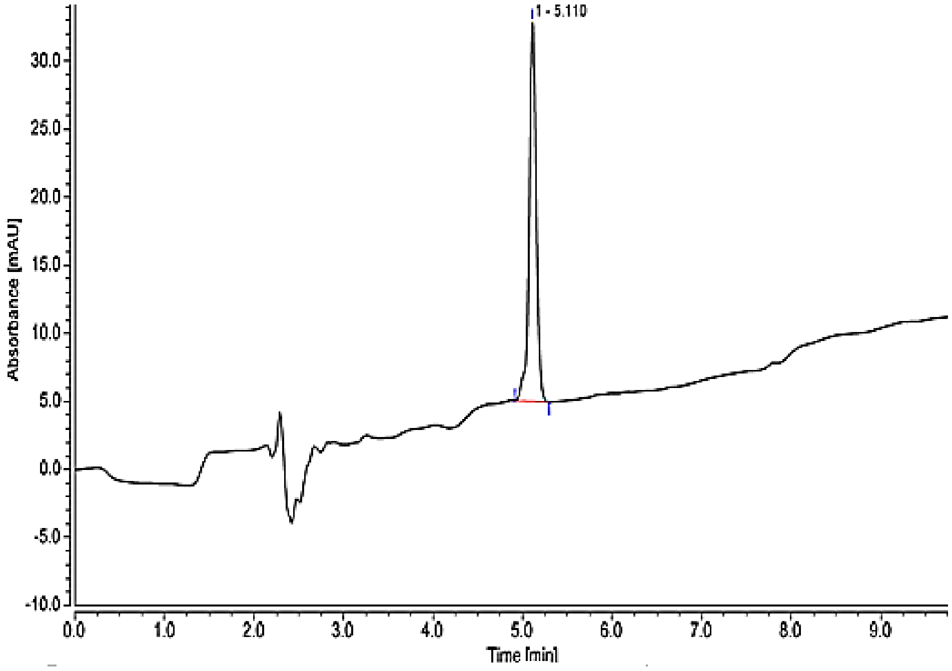

Figure S1. HPLC chromatogram of SV. Abbreviations: SV, sodium valproate.

Table S1. Prescreening study for formulation of SV-loaded SNVs.

Formula	Span 60 to EA ratio	Type of EA	Rotation speed (rpm)	EE %
**P1**	50:50	Cremophor RH	500	70.33±1.20
**P2**	60:40	Cremophor RH	500	82.30±0.98
**P3**	80:20	Cremophor RH	500	97.68±1.93
**P4**	50:50	Tween 80	500	67.22±1.41
**P5**	60:40	Tween 80	500	79.44±1.15
**P6**	80:20	Tween 80	500	95.53±1.46
**P7**	50:50	Brij 35	500	59.22±1.61
**P8**	60:40	Brij 35	500	69.23±1.25
**P9**	80:20	Brij 35	500	86.13±1.33
**P10**	50:50	Cremophor RH	1000	64.60±1.24
**P11**	60:40	Cremophor RH	1000	79.48±1.33
**P12**	80:20	Cremophor RH	1000	93.34±0.92
**P13**	50:50	Tween 80	1000	60.50±1.27
**P14**	60:40	Tween 80	1000	70.18±0.62
**P15**	80:20	Tween 80	1000	90.32±2.18
**P16**	50:50	Brij 35	1000	55.63±1.33

**P17**	60:40	Brij 35	1000	65.39±1.22
**P18**	80:20	Brij 35	1000	80.12±1.18
**P19**	50:50	Cremophor RH	1500	57.22±1.13
**P20**	60:40	Cremophor RH	1500	70.18±1.44
**P21**	80:20	Cremophor RH	1500	90.08±2.11
**P22**	50:50	Tween 80	1500	52.33±1.14
**P23**	60:40	Tween 80	1500	64.22±1.28
**P24**	80:20	Tween 80	1500	87.14±1.65
**P25**	50:50	Brij 35	1500	47.18±1.14
**P26**	60:40	Brij 35	1500	60.07±1.38
**P27**	80:20	Brij 35	1500	76.19±1.29

Notes: All formulations contained 8.3% SV; different values are expressed as mean ± SD (n = 3); 50:50 ratio involves using 50 mg Span 60 and 50 mg EA; 60:40 ratio involves using 60 mg Span 60 and 40 mg EA; 80:20 ratio involves using 80 mg Span 60 and 40 mg EA. Abbreviations: EE, entrapment efficiency; SV-loaded SNVs, Sodium valproate-loaded spanlastic nano-vesicles.

Table S2. Determination of the elasticity of SV-loaded SNVs and the corresponding niosomes.

Formula	PS before extrusion (nm)	PS after extrusion(nm)	DI
F8	222.9 ± 2.39	210.5 ± 1.88	22.57 ± 1.5
Niosomes	293.6 ± 1.33	44.93 ± 1.55	0.98 ± 0.03

Notes: F8, the optimized SV-loaded SNVs; the values are expressed as mean ± SD; n = 3. Abbreviations: EA, edge activator; DI, deformability index; PS, particle size.

Table S3. The correlation between the hair follicles number/cm^2 in the studied groups.

Duration	SV-loaded SNVs (n = 40)	Minoxidil (n = 40)	p-value
Pretreatment	17.9 ± 2.1	18.3 ± 2.75	0.358
1 month after treatment	17.2 ± 1.28	14.4 ± 1.98	0.047
2 month after treatment	16.76 ± 2.07	13.1 ± 2.51	0.031
3 month after treatment	19.45 ± 4.03	19.97 ± 4.18	0.593
4 month after treatment	26.27 ± 4.97	25.55 ± 5.12	0.328
5 month after treatment	29.19 ± 5.28	30.87 ± 6.11	0.314
6 month after treatment	31.58 ± 6.49	32.54 ± 6.39	0.286

Notes: P-value less than 0.05 indicate model terms are significant. Abbreviations: SV, sodium valproate; SNVs, spanlastic nanovesicles; P- value, probability value.

Table S4. The correlation between the hair shaft diameter (μm) in the studied groups.

Duration	SV-loaded SNVs (n = 40)	Minoxidil (n = 40)	p-value
Pretreatment	51.5 ± 5.27	50.7 ± 5.44	0.371
1 month after treatment	42.9 ±4.28	43.2 ± 4.52	0.482
2 month after treatment	43.18 ±3.98	43.9 ± 3.89	0.539
3 month after treatment	46.17 ± 4.03	47.27 ± 4.83	0.294
4 month after treatment	57.9 ± 6.94	56.35 ±5.83	0.271
5 month after treatment	59.36 ± 5.29	60.27 ± 4.39	0.287
6 month after treatment	62.4 ± 7.54	63.19 ± 7.49	0.483

Notes: P-value less than 0.05 indicate model terms are significant. Abbreviations: SV, sodium valproate; SNVs, spanlastic nanovesicles; P- value, probability value.

Table S5. The correlation between the hair density/cm^2 in the studied groups.

Duration	SV-loaded SNVs (n = 40)	Minoxidil	p-value

		(*n* = 40)	
Pretreatment	143.2 ± 10.11	146.04 ± 11.23	0.129
1 month after treatment	130.27 ± 12.28	131.94 ± 13.55	0.366
2 month after treatment	144.27 ± 18.31	142.07 ± 18.69	0.157
3 month after treatment	153.77 ± 21.62	155.39 ± 22.65	0.163
4 month after treatment	184.53 ± 20.59	186.28 ± 21.65	0.148
5 month after treatment	190.15 ± 23.77	193.27 ± 22.36	0.116
6 month after treatment	191.26 ± 24.72	197.71 ± 26.23	0.059

Notes: P-value less than 0.05 indicate model terms are significant. Abbreviations: SV, sodium valproate; SNVs, spanlastic nanovesicles; P- value, probability value.

Table S6. Side effects among the studied groups.

Side effects	SV-loaded SNVs (*n* = 40)	Minoxidil (*n* = 40)	P-value
Initial shedding	5 (12.5%)	32 (80%)	<0.001
Irritation	4 (10%)	12 (30%)	0.005
Facial hypertrichosis	0 (0%)	3 (7.5%)	0.128
Dandruff	2 (5%)	1 (2.5%)	0.322

Notes: P-value less than 0.05 indicate model terms are significant. Abbreviations: SV, sodium valproate; SNVs, spanlastic nanovesicles; P- value, probability value.
